# Colombian coffee tree leaves multispectral images dataset

**DOI:** 10.1016/j.dib.2025.111421

**Published:** 2025-02-21

**Authors:** Jorge Luis Aroca-Trujillo, Alexander Perez-Ruiz

**Affiliations:** Universidad Escuela Colombiana de Ingeniería Julio Garavito, Bogotá, D.C., Colombia

**Keywords:** Rust, Multiespectral, Dataset, Coffee, Hemileia vastatrix

## Abstract

In this work, a unique database of 6726 multispectral images of coffee leaves is presented. These images were captured in JPG format for the RGB photos and in TIF format for the five multispectral bands: blue, green, red, NIR and red edge, providing a detailed view of different wavelengths of the electromagnetic spectrum. Images in TIF format have a color depth of 16 bits per pixel, ensuring good quality.

The blue band (Band 1) captures light in the blue region of the spectrum, approximately 450 to 500 nm. The green band (Band 2) records light in the green region, approximately between 500 and 620 nm. The red band (Band 3) captures light in the red region, between 620 and 750 nm. The red-edge band (Band 4) lies between the red band and the NIR, and is sensitive to the transition between green vegetation and non-vegetation, around 840 nm. Finally, the near infrared band (Band 5) captures light in the near infrared region, between 750 and 900 nm.

For ease of identification, images are labeled as follows: if the image name ends in 0, it is an RGB image; if it ends in 1, it corresponds to the blue band; if it ends in 2, to the green band; if it ends in 3, to the red band; if it ends in 4, to the red-edge band; and if it ends in 5, to the near-infrared band.

The images show coffee leaves with and without lesions caused by the Hemileia vastatrix fungus, known as coffee rust. These samples were collected from Colombian coffee farms and the images were captured under controlled lighting conditions to ensure quality and consistency.

This database is an invaluable resource for precision agriculture research and early detection of crop diseases. With these 6726 images, researchers can use advanced image processing and machine learning techniques to identify differences between healthy leaves and those affected by rust. This can lead to the development of effective predictive models, enabling early detection and more efficient management of diseases in coffee plantations, optimizing production and reducing economic losses for farmers.

Specifications Table

**Table:** 

Subject	Data Article (Agricultural Sciences).
Specific subject area	*Agronomy and Crop Science.*
Type of data	*Images (.JPG and .TIF)*
Data collection	*The data collected consisted of 6726 images captured in a controlled environment at a distance of one meter using a multispectral camera equipped with 6 lenses. The images were stored in two different formats: JPG for the RGB images and TIF for the multispectral images for the blue, green, red, NIR and red-edge bands. The TIF format images have a color depth of 16 bits per* pixel*.*
Data source location	*Institution: Escuela Colombiana de Ingeniería Julio Garavito University* *City/Town/Region: Bogotá D.C.* Country: Colombia Latitude: 4.5983° * Longitude: 74.0051°.
Data accessibility	Repository name: *Coffe Rust* Data identification number: 10.34740/kaggle/ds/5644659 Direct URL to data: https://www.kaggle.com/ds/5644659 Instructions for accessing these data: *Data available free of charge to anyone with access to the Internet and the web server address provided.*
Related research article	*[**1**] Jorge Luis Aroca Trujillo, Alexander Pérez-Ruiz. “Technologies Applied in the Field of Early Detection of Coffee Rust Fungus Diseases: A Review.” Nongye Jixie Xuebao/Transactions of the Chinese Society of Agricultural Machinery 54.5 (2023).*

## 1 Value of the Data

• It provides multispectral images of coffee leaves that allow for a deeper understanding of the impact of the disease produced by the Hemileia vastatrix fungus on the physiology of the plant and the reduction of productivity. This drives the development of effective strategies to mitigate losses in coffee production in Colombia and the world.
• It facilitates the creation of predictive models based on machine learning for early detection of rust, allowing precise interventions such as the targeted application of fungicides, selective pruning and the optimization of agricultural practices.
• It fosters the development of universal tools to address phytosanitary problems in coffee and other crops, protecting an essential sector for thousands of rural families.
• Promotes a more sustainable agriculture and strengthens global productivity through research on plant health, nutritional deficiencies, and the management of environmental factors, consolidating resilient and efficient production.

## 2 Background

Rust disease can significantly reduce crop productivity, generating considerable economic losses for coffee growers [[1], [2], [3]]. Early detection and effective management are essential to mitigate the negative impact. Traditionally, rust identification has been carried out visually and manually, which in many cases results in an inefficient and costly practice, especially in large plantations. In addition, rust symptoms, such as leaf spots, are visible at an advanced stage of infection, which makes timely diagnosis difficult.

The limitations of traditional disease management have prompted new, more advanced tools that allow early and automatic detection of rust. In this context, RGB and multispectral image acquisition has become a promising technique in precision agriculture [[4], [5], [6]]. Multispectral imaging allows observing plant behavior at different wavelengths of the electromagnetic spectrum, which facilitates the identification of leaf anomalies that may not be visible in the visible light spectrum. By integrating data captured in various spectral bands, it is expected to develop more accurate and robust predictive models for the management of diseases affecting the coffee crop [7].

## 3 Data Description

**Fig. 1:**
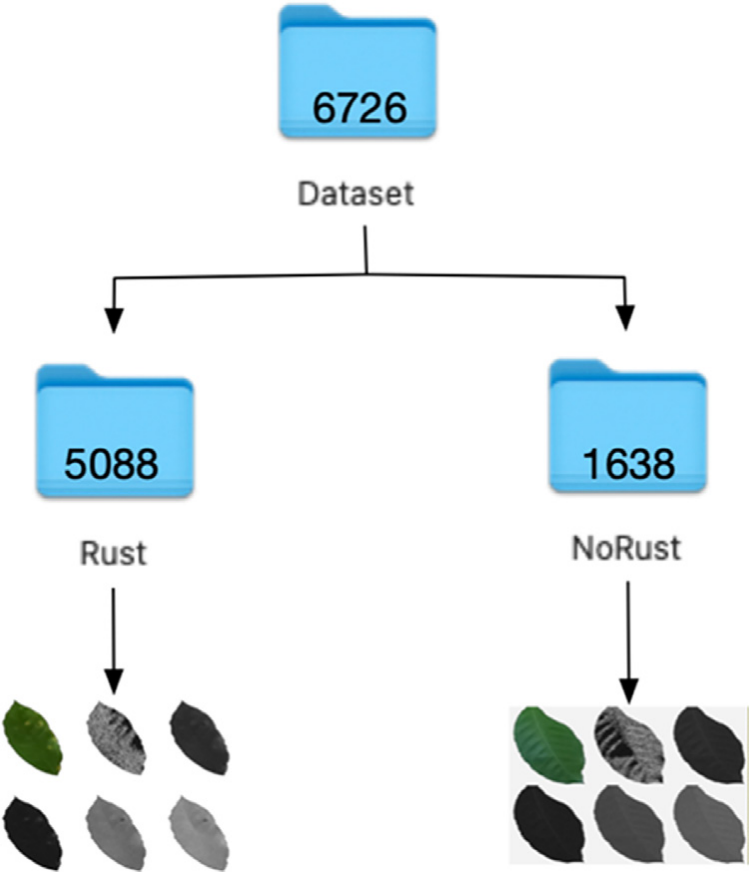
Image labeling.

The dataset is provided as a ZIP file. It consists of 6726 images of coffee leaves captured in two formats: JPG for the RGB images and TIF for the multispectral images, which are separated into two folders named Rust and NoRust (see Fig. 1). The images were taken under controlled conditions at a distance of one meter using a multispectral camera equipped with 6 lenses: one for RGB images and five for the blue, green, red, red-edge, and NIR spectral bands.) In addition to being taken under controlled conditions, the images are corrected by the illumination sensor present in the camera that adjusts the spectral values obtained in the images for any condition.

Each photo was taken with a resolution of 1600 × 1300 pixels, however, each image in the database is adjusted to the size of the leaf, so images of different sizes can be found. Additionally, the background of each image was removed through a mask to obtain the relevant information (coffee leaf), i.e., the background pixels have values equal to zero and in the case of multispectral images have a depth of 16 bits per pixel.

The image labeling system follows a specific convention to facilitate their identification: images ending in 0 are RGB, while those ending in 1 to 5 correspond to spectral bands (1 for blue, 2 for green, 3 for red, 4 for red edge, and 5 for NIR).

## 4 Experimental Design, Materials and Methods

A dataset has been created that incorporates healthy coffee leaves and leaves affected by the Hemileia vastatrix fungus [5,8]. The samples were collected in rural areas of coffee producing municipalities such as Garzón and Hobo, in the department of Huila, Colombia. In Garzón, the leaves come from three plantations located around the geographical coordinates 2°12′17.49 “N, 75°33′53.82 ‘W, while in Hobo, the samples come from a plantation near 2°31′10.6 ’N, 75°23′59.8 ”W.

It should be noted that Huila has more than 145.000 ha of coffee plantations, whose harvest represents 53 % of the regionʼs exports. This sector generates 101.000 direct jobs in the region, which indicates that 74 % of the rural population of the department depends on coffee growing, highlighting its economic and social importance in the region.

The images were taken in April, coinciding with moderate temperatures and high relative humidity typical of this time of year in the department of Huila, factors that favor the development of the Hemileia vastatrix fungus. At this date, the plantations had an average age of 2 years, corresponding to an early productive stage. Therefore, mainly young leaves with low infestations were collected; however, some leaves with a high level of infection were also identified.

This phenological phase of the plantations is particularly relevant, since young plants present specific physiological characteristics that can influence their susceptibility to diseases such as coffee rust. In addition, working with homogeneous crops in terms of age and harvest stage allows standardization of the data collected, promoting a more precise comparison between healthy and affected leaves. However, if the spread of the disease is not quickly controlled, it will cause premature leaf drop, negatively affecting the photosynthesis process of the plant and potentially reducing both the quantity and quality of the bean by up to 80 % [1].

These aspects not only enrich the quality of the data set, but also provide a solid basis for future research aimed at correlating crop age with rust susceptibility or developing optimized management strategies for critical production stages.

**Fig. 2:**
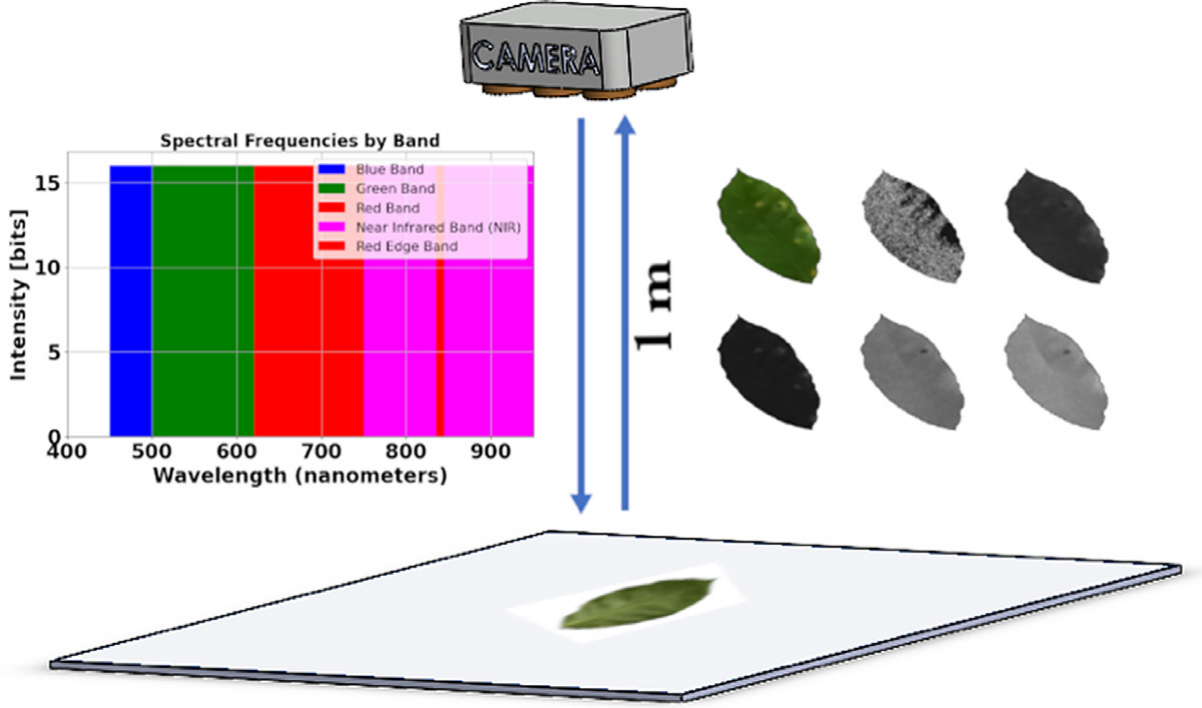
Image capture.

To capture the images, several coffee leaves were randomly collected from the crops. These leaves were immediately placed on a homogeneous background for photography. Subsequently, this background was removed and replaced by zero values in each pixel. The camera is positioned from a zenithal position (Fig. 2) and six images are captured: one RGB and five spectral bands.

The captured images are represented by an image composed of 3 color matrices that make up the RGB format (Red, Green and Blue) and five multispectral bands with bandwidths of:

• Blue band: Approximately between 450 and 500 nm [5].
• Green band: Approximately between 500 and 620 nm [5].
• Red band: Approximately between 620 and 750 nm [5].
• Near infrared (NIR) band: Approximately between 750 and 900 nm [5].
• Red-edge band: About 840 nanometers, located between the red and NIR bands, and is particularly useful for observing the transition between green vegetation and non-vegetation [5].

This database includes coffee leaves affected by the Hemileia vastatrix fungus (Rust) and by healthy leaves (NoRust), which makes it an ideal resource for training and validating machine learning models aimed at the automatic detection and classification of diseases in coffee crops. Within these categories are six images labeled as follows:

• RGB images: Ending in 0.
• Blue band (Band 1): They end in 1.
• Green band (Band 2): They end in 2.
• Red band (Band 3): They end in 3.
• Red border band (Band 4): They end in 4.
• Near infrared band - NIR (Band 5): End in 5.

**Fig. 3:**
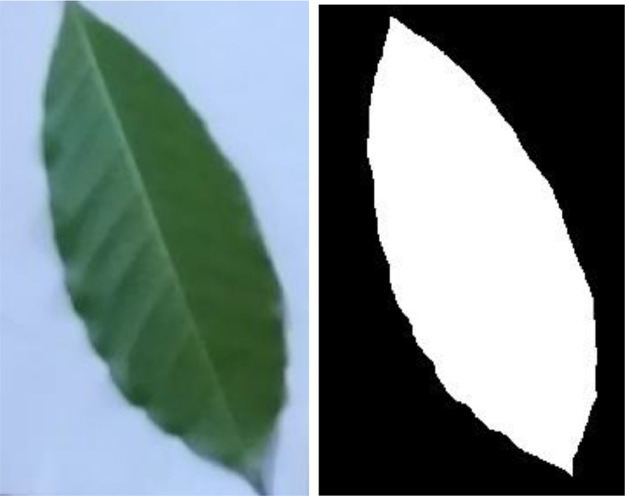
Original image and mask applied.

These multispectral bands allow the observation of different physiological properties of coffee leaves, which facilitates the detection of anomalies such as rust, which can alter the characteristics reflected in these spectra. This allows the development of computational algorithms for a comprehensive analysis of the impact of rust on leaves and provides a solid platform to advance agricultural research and the development of more efficient monitoring tools for coffee growers (Fig. 3).

**Fig. 4:**
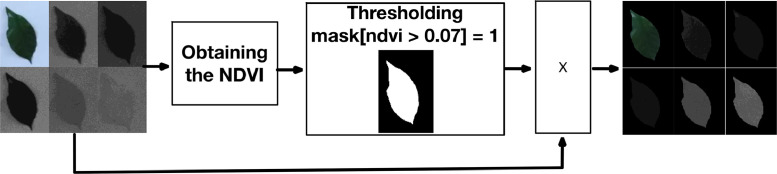
Image masking.

Once the segment of the image containing the coffee leaf, whose background is homogeneous, has been extracted, additional processing is carried out to eliminate this background. This step is carried out by multiplying the mask obtained from a thresholding of the NDVI spectral index with the RGB images and the five bands, as shown in Fig. 4.

**Fig. 5:**
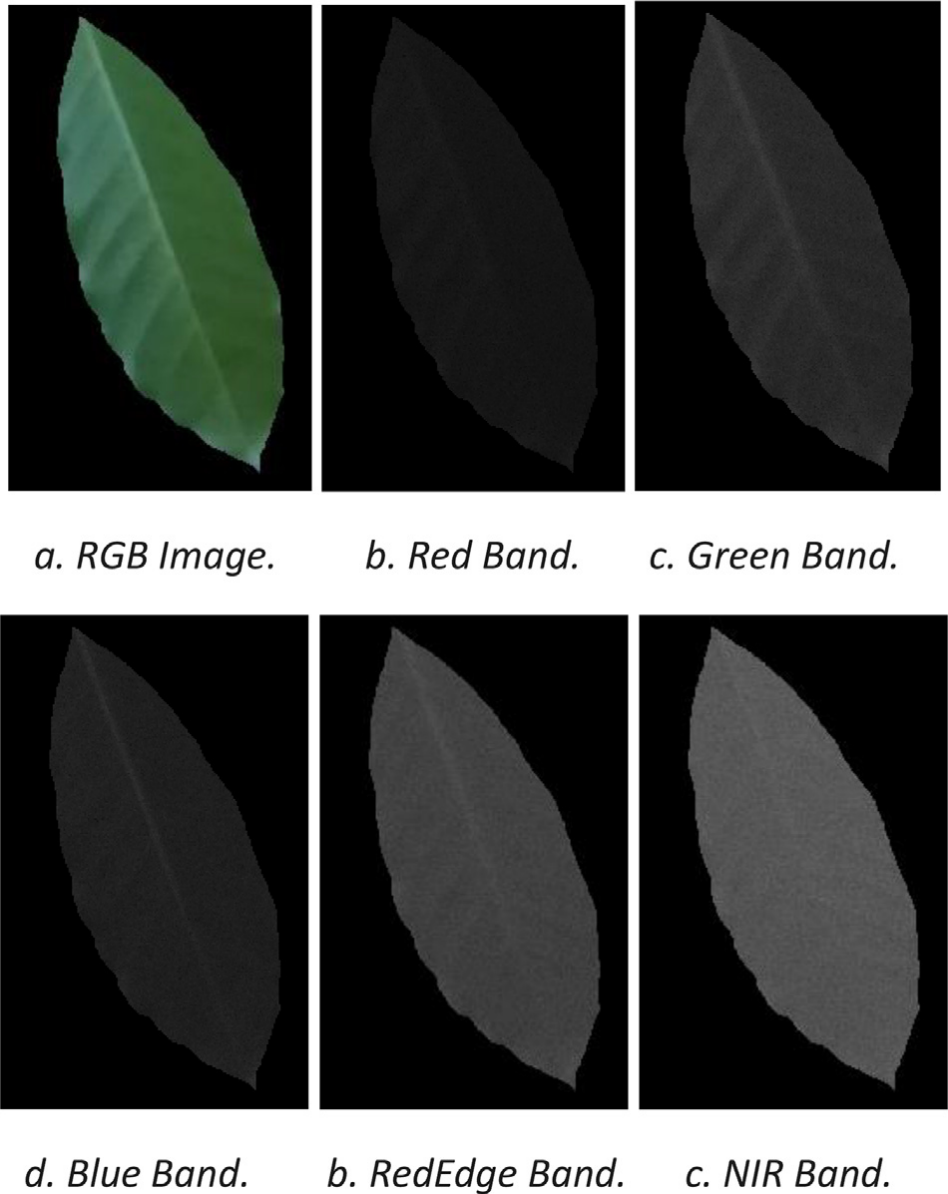
Images after passing through the mask.

The mask acts as a filter on the original image, assigning black or zero values to all areas corresponding to the background. In this way, only the coffee leaf remains visible in the image, while the background is reduced to a uniform color that does not interfere with further analysis (See Fig. 5).

The use of this mask not only allows the background to be removed, but also simplifies and improves the results of the computational analysis. By removing any distractions or visual noise from the environment, a cleaner image is obtained, which facilitates the subsequent detection of key features, such as areas affected by rust. This procedure is crucial to ensure that the analysis is performed accurately and focused only on the relevant aspects of the leaf. The figures above clearly illustrate how this masking process is carried out for effective background removal to provide noise-free images.

It is important to mention that the development of a tool based on classification models for the early detection of coffee rust promotes agricultural sustainability by allowing a more efficient management of the crop. This technology increases productivity by minimizing losses caused by the disease, strengthens the economic stability of coffee producing regions by protecting the income of producers, and promotes environmental care by optimizing the use of resources such as fungicides, reducing their environmental impact. Its implementation benefits both local communities and the global market, ensuring a balance between efficiency, economy and sustainability in the main coffee producing regions of the world.

## Limitations

Since the images were captured under controlled illumination conditions, it is important that further studies be carried out with caution, as these conditions may not be applicable in field scenarios where environmental variations are significant. However, it is relevant to note that the camera is equipped with an illumination sensor, which allows more accurate and reliable data to be obtained in any environment, considerably decreasing the need for additional processing.

Although multispectral images cover important bands such as blue, green, red, NIR and red edge, the lack of additional bands, such as ultraviolet or mid-infrared, limits the detection of certain patterns or key features for further diagnosis. Finally, the resolution of the images, while adequate for many purposes, may not be sufficient to identify small details of the disease, which could affect early detection of rust.

In addition, the database has a defined number of samples taken, which represents an important limitation in terms of diversity. This data set may not be representative of the wide variety of coffee varieties that exist in different regions and climatic conditions around the world.

## Ethics Statement

The authors have read and followed the ethical guidelines for publishing in Data in Brief, and they affirm that the present study does not involve human subjects, animal research, or data gathered from social media sites.

## CRediT Author Statement

**Jorge Luis Aroca Trujillo:** Conceptualization, Methodology, Investigation Data Curation, Writing - Original Draft; **Alexander Pérez Ruiz:** Conceptualization, Methodology, Investigation Data Curation, Writing - Original Draft.

## Data Availability

KaggleCoffee Rust (Original data)
